# Spending and Hospital Stay for Melanoma in Hunan, China

**DOI:** 10.3389/fpubh.2022.917119

**Published:** 2022-07-19

**Authors:** Xinchen Ke, Wenrui Lin, Daishi Li, Shuang Zhao, Mingliang Chen, Yi Xiao, Xiang Chen, Minxue Shen, Juan Su

**Affiliations:** ^1^Department of Dermatology, Xiangya Hospital, Central South University, Changsha, China; ^2^Hunan Engineering Research Center of Skin Health and Disease, Xiangya Hospital, Central South University, Changsha, China; ^3^Hunan Key Laboratory of Skin Cancer and Psoriasis, Xiangya Hospital, Central South University, Changsha, China; ^4^National Clinical Research Center of Geriatric Disorders, Xiangya Hospital, Central South University, Changsha, China; ^5^Department of Social Medicine and Health Management, Xiangya School of Public Health, Central South University, Changsha, China

**Keywords:** melanoma, Chinese hospitalization spending, length of stay, economic burden, surgery

## Abstract

**Objective:**

This study aimed to describe the economic burden of Chinese patients with melanoma in Hunan province of China, and to investigate the factors for hospitalization spending and length of stay (LOS) in patients undergoing melanoma surgery.

**Methods:**

Data was extracted from the Chinese National Health Statistics Network Reporting System database in Hunan province during 2017–2019. Population and individual statistics were presented, and nonparametric tests and quantile regression were used to analyze the factors for spending and LOS.

**Result:**

A total of 2,644 hospitalized patients with melanoma in Hunan were identified. During 2017–2019, the total hospitalization spending was $5,247,972, and out-of-pocket payment (OOP) was $1,817,869, accounting for 34.6% of the total expenditure. The median spending was $1,123 [interquartile range (IQR): $555–2,411] per capita, and the median LOS was 10 days (IQR: 5–18). A total of 1,104 patients who underwent surgery were further analyzed. The non-parametric tests and quantile regression showed that women were associated with less spending and LOS than men. In general, patients aged 46–65 and those with lesions on the limbs had higher hospitalization costs and LOS than other subgroups.

**Conclusion:**

Melanoma causes heavy economic burdens on patients in Hunan, such that the median spending is close to 60% of the averagely annual disposable income. Middle-aged men patients with melanoma on the limbs present the highest financial burden of melanoma.

## Introduction

Melanoma is a highly malignant tumor that originates from melanocytes. According to global cancer statistics 2018, melanoma of the skin ranked 21st among all cancers, respectively, taking up 1.6% (287,723) of all new cases and 0.6% (60,712) of death (1). The incidence of melanoma in East Asia has grown sharply from 1990 to 2019 (2). Melanomas are pretty heterogeneous across ethnicities. The main subtypes in Asians are acral melanoma and mucosal melanoma, while in Caucasians, melanomas are mainly associated with cumulative solar damage (3). Moreover, the prognoses of non-Caucasian patients with melanoma are worse than their counterparts (4). Although melanoma is a relatively rare cancer in China, the increasing incidence rate and the high fatality rate are nonnegligible (5). In China, the age-standardized incidence rate of melanoma was.9 per 100,000 in 2017, with a 110.3% increase in 1990. The disability-adjusted life-years (DALYs) of melanoma in China increased significantly by 13.6% rising annually from 2007 to 2017, with an age-standardized rate of 7.6 per 100,000 in 2017 (6). According to the CONCORD program, the 5-year survival rate of cutaneous melanoma (CM) in China was <60% (7).

To date, surgical resection is still an essential part of melanoma treatment (8). In China, most of the surgeries were performed on limbs since 42–58% of the patients suffered from acral lentiginous melanoma (9). The standard surgery procedure includes wide excision with or without sentinel node biopsy or lymph node dissection. Appearance and function of the extremities will be impaired if the surgery is not performed properly. Surgeries for advanced-stage melanoma present difficulties to dermatologists, as complex reconstructive procedures are required, imposing a heavy financial burden on patients and their families. After surgery, patients still need multiple hospitalizations for further disease assessment and follow-up treatment. In consideration of this, researchers have analyzed the relevant factors on the length of stay (LOS) and hospitalization costs in US adolescent and young adult patients with melanoma (10). Although hospitalization cost is one of the main burdens for patients with melanoma (11), few studies describe and analyze the economic features of melanoma hospitalization in China.

The costs of cancer treatments are unaffordable for many individuals and families, especially in developing countries. People with lower socioeconomic status often experience delayed healthcare, leading to a more advanced stage of disease and higher costs, and worse prognoses. Hence, the health insurance system plays a significant role in improving health equity and patients' prognoses by facilitating timely treatments. In recent years, many studies explored the impact of the Chinese health insurance system on LOS and the cost of colorectal cancer (11), lung cancer (12), and gastric cancer (13), but few investigated melanoma.

The current study aimed to describe the financial burden of melanoma and investigate the factors for LOS and costs for patients under surgery in Hunan. Hunan province locates in the middle reaches of the Yangtze River and south of Lake Dongting. The incidence and DALYs of melanoma in Hunan Province were among the highest in China (6). We collected melanoma inpatient data in Hunan province from January 2017 to December 2019 and estimated the hospitalization spending and out-of-pocket (OOP) payment for melanoma. We also extracted the data of patients who underwent surgery during this period and conducted a regression analysis of the relevant factors for LOS and hospitalization costs for the first time in China.

## Materials and Methods

### Data Source

The data source was the Chinese National Health Statistics Network Reporting System (CNHSNRS) in Hunan. Patients with a primary diagnosis of melanoma (ICD-10 Code: C43 or D03) were included. Since CNHSNRS was used to report hospitalization information of all diseases, the TNM stage and Breslow thickness were not recorded in the system. In our study, we extracted variables including the year of hospitalization (2017–2019), age (further divided into three groups ( ≤ 45, 46–65, and >65 years), sex, subsite (head and neck, limbs, trunk, and unspecified), surgery code, LOS, and costs (total, insurance, and OOP). We converted the currency unit from Chinese yuan (CNY) to US dollars (USD) according to the exchange rate in 2017 (1 CNY = 0.148 USD), and then adjusted hospitalization spending according to the 2017-2019 Hunan Consumer Price Index (CPI), based on the annual reports of the National Bureau of Statistics of China. We also obtained data on the per-capita annual disposable income of urban residents and farmers in Hunan Province in 2017 ($3,418.8).

### Statistical Methods

We used a Sankey diagram to describe the total costs by beneficiary characteristics (age, payer, and subsite). The study used a pyramid diagram to describe the distribution of costs by sex and age group.

Because surgery is a primary treatment for melanoma, patients were divided into surgical and non-surgical groups. Continuous variables were expressed as mean ±*SD*, while categorical data were presented as count (proportion, %). Student *t*-tests and Chi-square tests were used to compare group differences.

Data of patients receiving surgery were further selected for the analyses of LOS and cost. By performing the Kolmogorov-Smirnov test, we found that LOS and cost were of skewed distribution (*P* < 0.05), and median with interquartile range (IQR) were used to describe the data. We used the Mann-Whitney U test or the Kruskal-Wallis test to compare subgroups and used ordinary least squared (OLS) regression and quantile regression (QR) to explore the factors for spending and LOS.

Data management and analyses were processed with R version 4.0.3. The parameters of the QR model were estimated at the 10th, 25th, 50th, 75th, and 90th percentiles of cost or LOS using the package “quantreg”. The Pyramid diagram was plotted using package “ggplot2”. The Sankey diagram was plotted using a commercial online tool (https://dycharts.com).

## Results

### Spending at the Population Level

**Figure 1:**
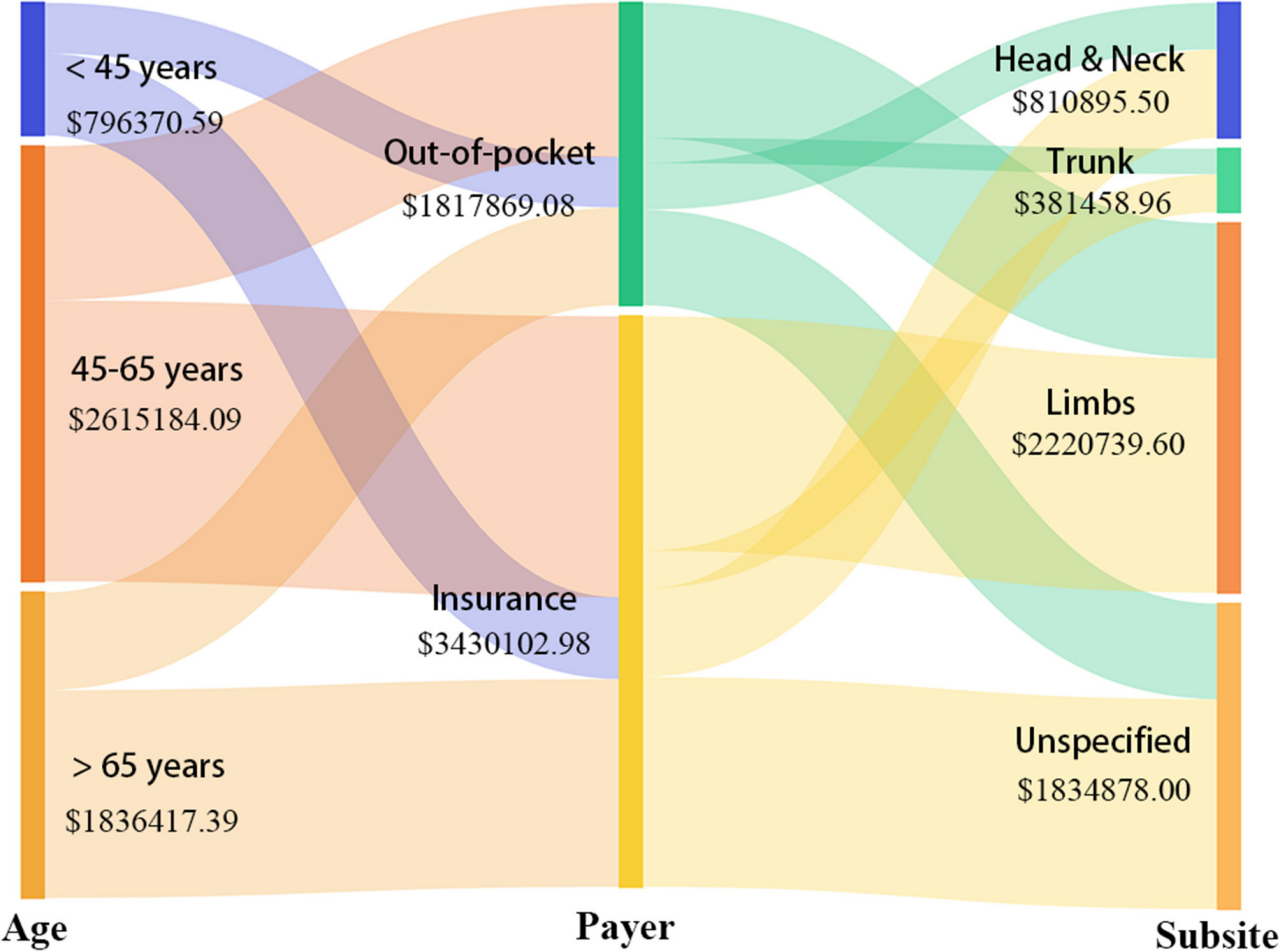
Visualization of total costs for beneficiaries with different characteristics (age, payer, and subsite).

**Figure 2:**
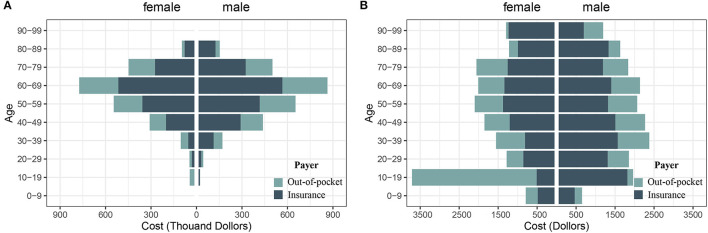
Pyramid diagram to describe the distribution of costs by sex and age group. **(A)** Cost at population level; **(B)** Cost at individual level.

During 2017–2019, the total hospitalization spending for 2,644 patients with melanoma was $5,247,972 and OOP was $1,817,869. Figure 1 displays the composition of hospitalization spending by age, payer, and subsite, and melanoma on limbs accounted for the highest proportion of spending. Figure 2 shows the costs across age and sex groups. The 60–69 age group accounted for the largest portion of total expenditure. The proportion of OOP varied across age groups, such that child and adolescent patients paid more OOP money.

### Spending and LOS at the Patient Level

A total of 2,644 hospitalized records of patients with melanoma in Hunan province from 2017 to 2019 were extracted (Table 1), including 1,242 (47%) women and 1,402 (53%) men. The mean age was 59.60 ± 14.94 years. The number of patients was 780 (29.5%), 896 (33.9%), and 968 (36.6%) in 2017, 2018, and 2019, respectively. 480 (18.1%) patients had melanoma on head & neck, 892 (33.7%) on limbs, and 216 (8.2%) on trunk. In 1,056 (39.9%) patients, the location of the skin lesions was unspecified. The median spending was $1,123 [interquartile range (IQR): $555–2,411] per capita, and the median LOS was 10 days (IQR: 5–18).

**Table 1:** Baseline characteristics of Hunan melanoma inpatients from 2017–2019.

		**Surgery**		***P***
**Variables**	**Total**	**No**	**Yes**	
All	2,644 (100)	1,540 (100)	1,104 (100)	
**Age, mean (SD)**	59.60 (14.94)	60.39 (14.01)	58.50 (16.09)	0.001
**Age, n (%)**				0.002
≤ 45	417 (15.8)	214 (13.9)	203 (18.4)	
46–65	1,228 (46.4)	713 (46.3)	515 (46.6)	
>65	999 (37.8)	613 (39.8)	386 (35.0)	
**Sex, n (%)**				0.308
Male	1,402 (53.0)	830 (53.9)	572 (51.8)	
Female	1,242 (47.0)	710 (46.1)	532 (48.2)	
**Year, n (%)**				0.429
2017	780 (29.5)	440 (28.6)	340 (30.8)	
2018	896 (33.9)	533 (34.6)	363 (32.9)	
2019	968 (36.6)	567 (36.8)	401 (36.3)	
***In situ***, **n (%)**				<0.001
Non-*in situ*	2,515 (95.1)	1,486 (96.5)	1,029 (93.2)	
*In situ*	129 (4.9)	54 (3.5)	75 (6.8)	
**Subsite, n (%)**				<0.001
Head and neck	480 (18.1)	254 (16.5)	226 (20.5)	
limbs	892 (33.7)	413 (26.8)	479 (43.4)	
Trunk	216 (8.2)	133 (8.6)	83 (7.5)	
Unspecified	1,056 (39.9)	740 (48.1)	316 (28.6)	

The results of subsequent analysis on LOS and spending in melanoma surgery patients are displayed in Table 2. A total of 1,104 patients underwent surgery, with a median cost of $2,088.8 (IQR: $901.1 to $3,937.6) and a median LOS of 14 days (IQR: 8 to 22). According to the rank-sum tests, higher costs were observed in the 46–65 age group, men patients, non-in-situ melanoma, and limbs subsite (*P* < 0.05). For LOS, significant differences were observed across groups of age, sex, year, surgery level, insurance, and subsite.

**Table 2:** Baseline characteristics of melanoma patients undergoing surgery in Hunan during 2017–2019.

**Variables**	***N* (%)**	**Cost (2017 US$) ** **(Median, interquartile range)**	***p***	**LOS (days)** ** (Median, interquartile range)**	***P***
**All**		2088.82 (901.13,3937.64)		14.0 (8.0,22.0)	
**Age**			<0.001		<0.001
≤ 45	203 (18.4)	1,679.91 (580.40, 3,572.73)		11.0 (5.0,19.0)	
46–65	515 (46.6)	2,644.29 (1,063.10, 4,348.39)		15.0 (9.0,24.0)	
>65	386 (35.0)	1,749.35 (913.11, 3,591.30)		14.0 (9.0,22.0)	
**Sex**			0.002		<0.001
Male	572 (51.8)	2,306.47 (1,011.77, 4,274.26)		15.0 (9.0,23.0)	
Female	532 (48.2)	1,872.00 (828.11, 3,646.53)		13.0 (8.0,21.0)	
**Year**			0.163		0.003
2017	340 (30.8)	2,006.31 (866.31, 4,236.89)		15.0 (9.0,26.0)	
2018	363 (32.9)	2,241.05 (1,049.16, 4,043.96)		15.0 (9.0,22.0)	
2019	401 (36.3)	2 007.03 (747.85, 3,669.53)		12.0 (8.0,21.0)	
***In situ***			<0.001		<0.001
Non-*in situ*	1,029 (93.2)	2,371.73 (1,019.13, 4,099.43)		14.0 (9.0,23.0)	
*In situ*	75 (6.8)	577.34 (351.15, 1,328.39)		5.0 (4.0,10.0)	
**Subsite**			<0.001		<0.001
Head and neck	226 (20.5)	1,330.58 (524.72, 2,895.09)		10.0 (5.0,14.0)	
limbs	479 (43.4)	3,089.82 (1,496.31, 4,730.67)		17.0 (10.0,25.0)	
Trunk	83 (7.5)	1,803.70 (902.74, 3,435.41)		12.0 (7.0,20.5)	
Unspecified	316 (28.6)	1,582.64 (630.81, 3,479.57)		14.0 (8.0,23.0)	

### Factors for Spending on Surgery Patients

**Figure 3:**
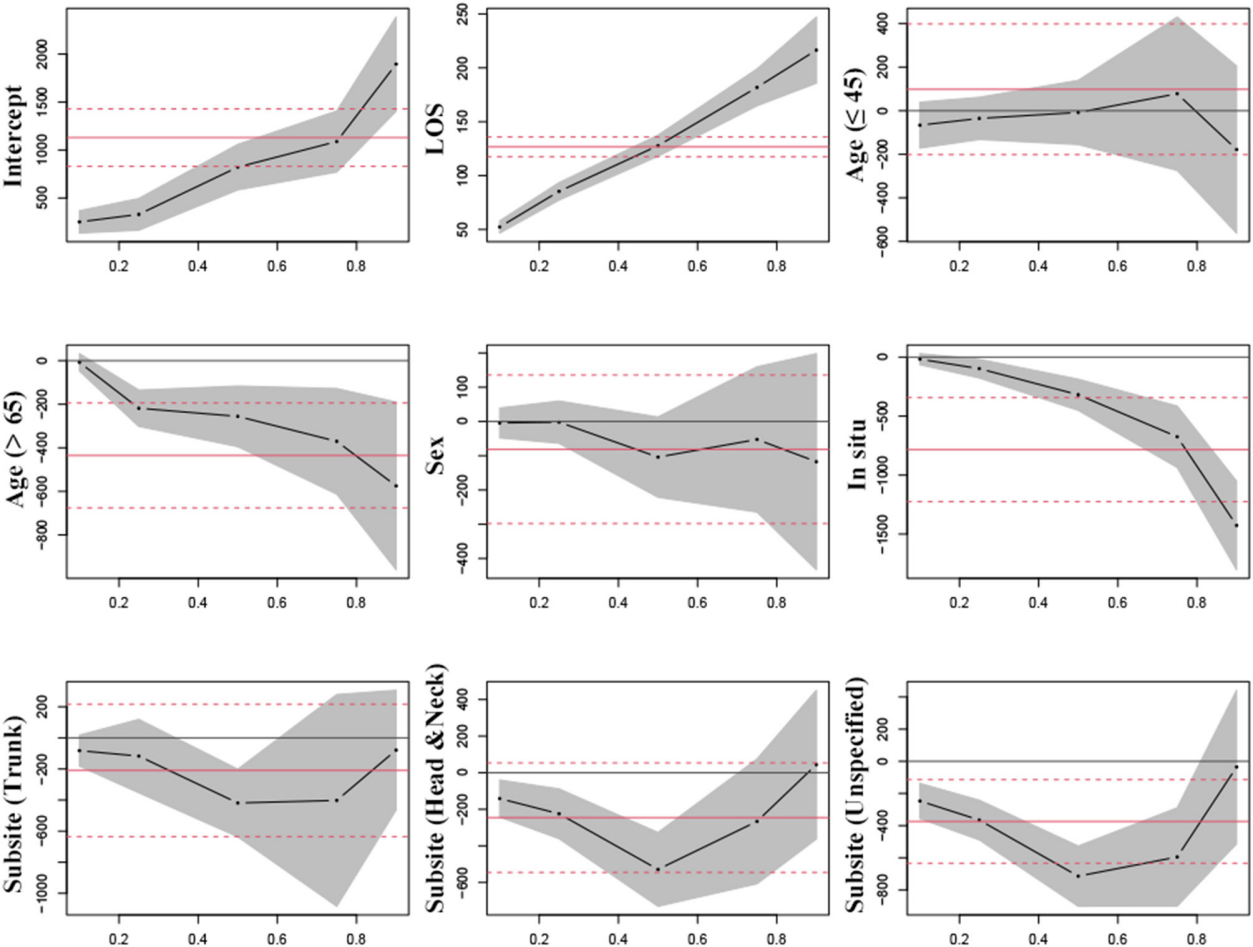
Estimated parameters for hospitalization costs in patients with melanoma undergoing surgery by quantile regression versus linear regression. Black lines signify the point estimates by quantile regression at 0.1, 0.25, 0.5, 0.75, and 0.9 quantiles (gray bands signify 95% confidence intervals); and red lines signify the point estimates by linear regression (red dash lines signify 95% confidence intervals).

Table 3 shows the QR coefficients across quantiles of costs. A correlation between LOS and hospitalization costs was observed, with effect sizes ranging from $52.31 (10th quantile) to $216.42 (90th quantile), and OLS presented an average effect size of $126.7 per day (Figure 3). Sex showed no significant effect on hospitalization cost across all quantiles. Patients with melanoma *in situ* had significantly lower costs for most quantiles. Besides, the costs in patients over 65 were lower than those aged 46–65 years, as a trend was identified by QR varying from $-218.47 at the 25th percentile to $-370.34 at the 75th percentile of spending. Patients with melanoma on limbs had higher costs than other sites in 50th and lower percentiles. Figure 3 shows the patterns of the changes in the coefficients across the quantiles of cost.

**Table 3:** Quantile regression versus linear regression for the hospitalization costs of patients with melanoma undergoing surgery in Hunan.

**Variables**	**OLS**	**10th Percentile**	**25th Percentile**	**50th Percentile**	**75th Percentile**	**90th Percentile**
**LOS (days)**	126.70^***^ (5.55)	52.31^***^ (3.44)	85.35^***^ (4.91)	127.74^***^ (5.95)	181.82^***^ (10.37)	216.42^***^ (18.66)
**Age**							
≤ 45	98.43 (182.46)	−66.46 (64.05)	−35.44 (58.94)	−8.08 (89.60)	77.92 (214.04)	−178.20 (233.25)
46–65	Ref.	Ref.	Ref.	Ref.	Ref.	Ref.
>65	−435.01^***^ (146.29)	−7.60 (24.21)	−218.47^***^ (50.60)	−255.58^***^ (84.61)	−370.34^**^ (147.30)	−574.89^**^ (233.91)
**Sex**							
Male	Ref.	Ref.	Ref.	Ref.	Ref.	Ref.
Female	−81.30 (131.79)	−4.32 (26.51)	−2.06 (37.50)	−104.10 (71.24)	−52.48 (128.94)	−117.39 (191.76)
***In situ***							
Non-*in situ*	Ref.	Ref.	Ref.	Ref.	Ref.	Ref.
*In situ*	−784.28^***^ (268.06)	−17.49 (28.50)	−97.46^**^ (48.08)	−319.05^***^ (80.24)	−674.96^***^ (158.24)	−1,426.55^***^ (227.96)
**Subsite**							
Head and neck	−245.58 (182.08)	−141.25^**^ (61.39)	−224.53^***^ (82.60)	−528.64^***^ (122.53)	−266.27 (207.29)	44.17 (247.06)
limbs	Ref.	Ref.	Ref.	Ref.	Ref.	Ref.
Trunk	−209.16 (259.00)	−82.17 (60.50)	−117.25 (143.52)	−419.12^***^ (132.16)	−402.44 (414.20)	−78.67 (234.08)
Unspecified	−374.06^**^ (158.00)	−246.84^***^ (66.50)	−365.23^***^ (74.87)	−714.45^***^ (113.81)	−594.90^***^ (185.70)	−35.59 (290.21)

*Standard errors in parentheses*.

** *p < 0.05*,

*** *p < 0.01*.

*LOS: length of stay, OLS: ordinary least squares*.

### Factors for LOS in Surgery Patients

**Figure 4:**
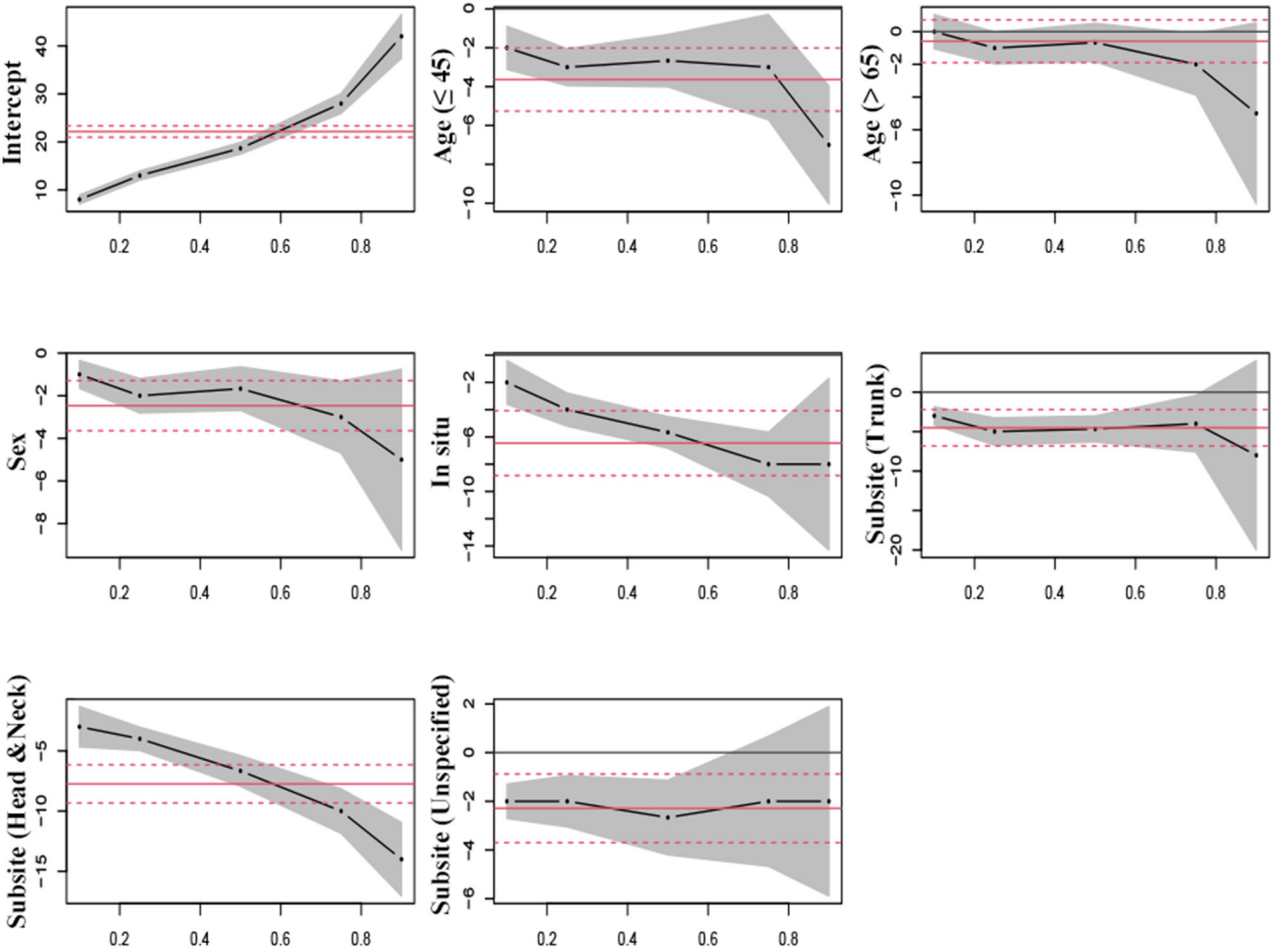
Estimated parameters for length of hospital stay in patients with melanoma undergoing surgery by quantile regression versus linear regression. Black lines signify the point estimates by quantile regression at 0.1, 0.25, 0.5, 0.75, and 0.9 quantiles (gray bands signify 95% confidence intervals); and red lines signify the point estimates by linear regression (red dash lines signify 95% confidence intervals).

Table 4 shows the QR coefficients across quantiles of LOS. LOS in women patients was significantly lower than in men at each quantile and on average (−2.47 days) according to OLS. Besides, younger patients ( ≤ 45) had a shorter LOS than middle-aged patients (46–65), with differences of −2, −3, −2.67, and −7 days at 10th, 25th, 50th, and 90th quantiles, respectively, and 3.63 days on average. Patients with melanoma *in situ* stayed fewer days across quantiles, while spending for melanoma on limbs was associated with a longer LOS in 50th and lower quantiles. Figure 4 shows the patterns of the changes in the coefficients across the quantiles of LOS.

**Table 4:** Quantile regression versus linear regression for LOS of patients with melanoma undergoing surgery in Hunan.

**Variables**	**OLS**	**10th Percentile**	**25th Percentile**	**50th Percentile**	**75th Percentile**	**90th Percentile**
**Age**							
≤ 45	−3.64^***^ (0.99)	−2.00^***^ (0.67)	−3.00^***^ (0.57)	−2.67^***^ (0.82)	−3.00^*^ (1.64)	−7.00^***^ (1.85)
46–65	Ref.	Ref.	Ref.	Ref.	Ref.	Ref.
>65	−0.59 (0.80)	0.00 (0.63)	−1.00^*^ (0.59)	−0.67 (0.71)	−2.00^*^ (1.14)	−5.00 (3.37)
**Sex**							
Male	Ref.	Ref.	Ref.	Ref.	Ref.	Ref.
Female	−2.47^***^ (0.71)	−1.00^**^ (0.39)	−2.00^***^ (0.49)	−1.67^***^ (0.62)	−3.00^***^ (1.03)	−5.00^*^ (2.58)
***In situ***							
Non-*in situ*	Ref.	Ref.	Ref.	Ref.	Ref.	Ref.
*In situ*	−6.46^***^ (1.45)	−2.00^**^ (0.97)	−4.00^***^ (0.73)	−5.67^***^ (0.70)	−8.00^***^ (1.42)	−8.00^**^ (3.82)
**Subsite**							
Head and neck	−7.74^***^ (0.96)	−3.00^***^ (1.01)	−4.00^***^ (0.58)	−6.67^***^ (0.77)	−10.00^***^ (1.12)	−14.00^***^ (1.85)
limbs	Ref.	Ref.	Ref.	Ref.	Ref.	Ref.
Trunk	−4.51^***^ (1.40)	−3.00^***^ (0.69)	−5.00^***^ (1.05)	−4.67^***^ (1.00)	−4.00^*^ (2.17)	−8.00 (7.29)
Unspecified	−2.29^***^ (0.86)	−2.00^***^ (0.43)	−2.00^***^ (0.64)	−2.67^***^ (0.93)	−2.00 (1.62)	−2.00 (2.36)

*Standard errors in parentheses*.

* *p < 0.1*,

** *p < 0.05*,

*** *p < 0.01*.

*LOS: length of stay, OLS: ordinary least squares*.

## Discussion

Our study described the LOS and hospitalization spending for melanoma in China. Melanoma causes considerable economic burdens on patients, such that the median spending is close to 60% of the averagely annual disposable income. Middle-aged men patients with melanoma on the limbs present the highest financial burden of melanoma.

Surgery patients aged 46–65 years had the highest costs. Compared to the elderly (>65 years old), middle-aged patients are in better health conditions, and they are likely to receive more aggressive treatment options to achieve better prognoses, resulting in higher expenditures. In contrast, younger patients need fewer LOS than middle-aged patients, owing to faster postoperative recovery and fewer postoperative complications.

Surgery patients who are women had fewer hospital stays and less spending than men, and this was consistent with the study by Buja et al. (14). A previous study (15) found that women patients tended to have a thinner Breslow thickness and were less likely to present ulcerated lesions or lymph node invasion. They also found that melanoma was diagnosed at an earlier stage in women. Even at the same TNM stage, women have a lower chance of metastasis and a significantly better prognosis than men (16, 17). Therefore, men patients are likely to undergo more complex procedures such as extended excision and sentinel lymph node biopsy. Besides, according to Lyth et al. (18), melanoma-related costs per patient-year increased as the clinical stage increased. The average annual cost for stage IV patients is 10 times higher than for stage I patients. These may contribute to the sex differences in LOS and costs.

Melanoma on limbs was associated with the highest LOS and spending. Since a large proportion of melanoma on limbs is acral melanoma, there are two possible explanations for the result. On the one hand, acral melanoma tends to be more aggressive, resulting in a wider range of surgical resections and a larger likelihood of sentinel lymph node biopsy. On the other hand, the functional reconstruction and nerve repair involved in extremity surgeries are more complicated than the trunk, head, and neck surgery.

Suffering from melanoma could be a devastating condition for any individual from economic, family, and social perspectives. Due to the lack of knowledge of melanoma and the fear of bringing a significant financial burden to the family, many patients did not seek timely medical help after a suspicious skin lesion was present. According to a study of 357 patients (19), 91.6% of Chinese patients with melanoma were at clinical stage II or above at presentation. The patient's delay in seeking medical help is one of the crucial reasons. Our study found that the median hospitalization costs for melanoma in surgery patients were 60% of the annual disposable income per capita in Hunan Province in 2017, and this may cause catastrophic medical expenditure if health insurance is not available. Nevertheless, China's health insurance reform has been successfully carried out for more than 10 years, and most residents have basic health insurance to partially reimburse the medical cost (20). We observed an average proportion of OOP of 35%, indicating that the cost of melanoma is affordable at the societal level yet still brings an economic burden to individual patients and families.

The study has limitations. First, the CNHSNRS database records hospitalizations for all diseases, such that melanoma-specific clinical information was not available. Second, the quality and completeness of data varied across participating hospitals, and inaccurate and missing information inevitably led to biases. Third, since melanomas on limbs share the same ICD code (such as melanoma of the foot and leg), the specific site of the lesion could not be identified. Last, in recent years, neoadjuvant therapy represented by immunotherapy and targeted therapy plays an essential role in treating advanced melanoma. More and more medications, such as Pembrolizumab and Dabrafenib plus Trametinib, were approved by the National Medical Products Administration of China in 2018. However, due to issues of drug supply, many patients purchased these drugs beyond the hospital. Therefore, we could not determine this part of spending through the CNHSNRS and may underestimate the economic burden of patients.

## Conclusion

By analyzing the hospitalization data of melanoma in Hunan province during 2017–2019, we preliminarily described the economic burdens at both population and individual levels and identified that middle-aged men with melanoma on limbs had the highest spending and LOS among subgroups of patients. Owing to the rapid development of treatments for advanced melanoma, it is necessary to evaluate the treatments from a health and economic perspective as well as to promote secondary prevention of melanoma to improve patient prognosis and reduce economic burden.

## Data Availability Statement

The raw data supporting the conclusions of this article will be made available by the authors, without undue reservation.

## Author Contributions

XK, WL, MS, and JS: study concepts and study design. WL, DL, SZ, and MS: data acquisition, analysis, interpretation, and statistical analysis. XK, MC, and YX: manuscript preparation and editing. XK, WL, XC, MS, and JS: manuscript review. All authors read and approved the final manuscript.

## Funding

This work was supported by the National Natural Science Foundation of China (Grant Nos. 81974478 and 82173009), the Project of Intelligent Management Software for Multimodal Medical Big Data for New Generation Information Technology, and the Ministry of Industry and Information Technology of People's Republic of China (TC210804V).

## Conflict of Interest

The authors declare that the research was conducted in the absence of any commercial or financial relationships that could be construed as a potential conflict of interest. The handling editor RH declared a shared affiliation with the authors at the time of review.

## Publisher's Note

All claims expressed in this article are solely those of the authors and do not necessarily represent those of their affiliated organizations, or those of the publisher, the editors and the reviewers. Any product that may be evaluated in this article, or claim that may be made by its manufacturer, is not guaranteed or endorsed by the publisher.
